# Red Blood Cell Antioxidant State in Fanconi Anemia: The Highlighted Roles of Pi-Class Glutathione S-Transferase and Glutathione Peroxidase

**DOI:** 10.3390/antiox14101150

**Published:** 2025-09-23

**Authors:** Cláudia Oliveira, Ricardo Jorge Dinis-Oliveira, Félix Carvalho, Paula Jorge, Beatriz Porto

**Affiliations:** 1 Cytogenetics Laboratory, Department of Microscopy, ICBAS—School of Medicine and Biomedical Sciences, University of Porto, 4050-313 Porto, Portugal; csoliveira@icbas.up.pt (C.O.); pmjorge@icbas.up.pt (P.J.); 2 UMIB—Unit for Multidisciplinary Research in Biomedicine, ICBAS—School of Medicine and Biomedical Sciences, University of Porto, 4050-313 Porto, Portugal; 3 ITR—Laboratory for Integrative and Translational Research in Population Health, ICBAS—School of Medicine and Biomedical Sciences, University of Porto, 4050-313 Porto, Portugal; 4 Laboratory i4HB—Institute for Health and Bioeconomy, CESPU—University Institute of Health Sciences, 4585-116 Gandra, Portugal; 5 UCIBIO—Research Unit on Applied Molecular Biosciences, Translational Toxicology Research Laboratory, CESPU—University Institute of Health Sciences, 4585-116 Gandra, Portugal; 6 Department of Public Health and Forensic Sciences and Medical Education, Faculty of Medicine, University of Porto, 4200-319 Porto, Portugal; 7 Associate Laboratory i4HB, Institute for Health and Bioeconomy, University of Porto, 4050-313 Porto, Portugal; 8 UCIBIO—Applied Molecular Biosciences Unit, Laboratory of Toxicology, Faculty of Pharmacy, University of Porto, 4050-313 Porto, Portugal

**Keywords:** Fanconi anemia, red blood cells, oxidative stress, GSTP1, GPx

## Abstract

Fanconi anemia (FA) is a rare bone marrow failure disorder characterized at the cellular level by hypersensitivity to alkylating agents, such as diepoxybutane (DEB), and redox imbalance. Alterations in red blood cells (RBCs), which play a key role in systemic antioxidant defense, are among the earliest changes in FA, consistent with an oxidative stress (OS) profile. Previous studies about antioxidant activity in RBCs from these patients are scarce and inconsistent. This study aimed to better understand the antioxidant profile in RBCs from FA patients carrying the homozygous *FANCA* c.295C>T variant. Glutathione content and the activities of catalase, superoxide dismutase, glutathione peroxidase (GPx), and Pi-class glutathione S-transferase (GSTP1) were quantified, both at baseline and after culture with and without DEB, in RBCs from FA patients, FA carriers, and controls. At baseline, FA RBCs displayed significantly reduced catalase activity, whereas GPx and GSTP1 activities were significantly increased, suggesting an OS preconditioning state, not observed in RBCs from FA carriers and controls. Under culture and DEB exposure, FA RBCs exhibited a significant decline in both GSTP1 and GPx activities, contrary to controls. These new findings highlight a key role of GSTP1 and GPx activities in baseline antioxidant defense, severely compromised in case of increased OS toxicity.

## 1. Introduction

Fanconi Anemia (FA) is a rare, genetically and clinically heterogeneous disorder, caused by biallelic pathogenic variants in one of the 22 FA genes so far characterized (*FANC-A* to *-W*) that encode proteins functioning in a common signaling pathway that controls the maintenance of genomic stability [1,2]. Clinically, FA patients may present diverse congenital anomalies, progressive bone marrow failure (BMF), and an increased predisposition to cancer, in particular acute myeloid leukemia and head and neck squamous cell carcinoma [3,4,5]. Despite the genetic and clinical variability, at the cellular level, all FA patients exhibit chromosome instability (CI), particularly in response to alkylating agents, such as 1,2,3,4-Diepoxybutane (DEB), which constitutes a unique and specific diagnostic marker [6]. This cellular phenotype reflects a reduced capacity of FA cells to counteract oxidative stress (OS)-related toxicity, already demonstrated by increased lipid peroxidation, free iron levels, reactive oxygen species (ROS) overproduction, mitochondrial dysfunction, and glutathione (GSH) depletion [7,8,9,10,11]. This incapacity contributes to bone marrow failure and to the broader clinical spectrum of FA, including ineffective erythropoiesis, cytopenia, increased tissue vulnerability, and a higher risk of infection and malignancy [12].

Red blood cells (RBCs), the most abundant cells in the human organism, play a crucial role in systemic antioxidant defense. They are highly specialized for their primary function, i.e., O_2_ transport from the lungs to tissues via hemoglobin (Hb), which inevitably exposes them to extremely high O_2_ levels, making them particularly exposed to pro-oxidant conditions [11,13]. To counteract this, RBCs rely on a complex antioxidant defense system that integrates enzymatic mechanisms (catalase, superoxide dismutase (OD)], glutathione peroxidase (GPx), and glutathione *S*-transferase (GST) isoenzymes) and non-enzymatic components, particularly reduced GSH [14,15,16]. This system not only preserves RBC integrity but also allows the enzymes to function as mobile scavengers, providing systemic protection against oxidative insults to other cells and tissues [11,13,14,15,16]. Among GST isoenzymes, Pi-class GST (GSTP1) is of particular interest. As a phase II detoxification enzyme, it conjugates GSH to electrophiles, aldehydes derived from lipid peroxidation, and ROS. While this activity is protective, it may also deplete intracellular GSH and disturb coordination with other antioxidant defenses [17,18]. The first direct evidence of an association between the FA pathway and OS was shown by the interaction of FANCC protein with GSTP1 and Nicotinamide Adenine Dinucleotide Phosphate (NADPH) cytochrome P450 reductase. [19,20]. However, no further studies exploring GSTP1 activity in FA RBCs were performed.

Alterations in RBC antioxidant defenses are among the earliest detectable changes in many OS-related pathologies, including FA. While early studies reported SOD deficiencies in FA RBCs, subsequent findings have been inconsistent, with some showing normal SOD and GSH levels but increased MnSOD, CAT, and GPx activities, likely reflecting a compensatory response to chronic pro-oxidant states [21,22]. In addition to biochemical alterations, morphological changes in FA RBCs have been linked to redox imbalance, leading to alterations in plasticity and deformation-associated functions [23,24].

FA pathogenesis involves a complex interplay among OS, inflammation, mitochondrial dysfunction, and impaired intracellular signaling, all contributing to BMF and disease progression [7,9,10,12]. Efforts to counteract redox imbalance in FA have included the use of antioxidant compounds, both in experimental models and clinical settings [25]. For instance, N-acetylcysteine (NAC) and α-lipoic acid (ALA) have been shown to reduce CI in primary lymphocytes from FA patients [26,27]. More recently, quercetin, a natural flavonoid with broad antioxidant and anti-inflammatory properties, has gained attention as a potential therapeutic in FA. Beyond scavenging ROS, quercetin can inhibit lipid peroxidation, stabilize membranes, and activate cytoprotective pathways such as Nrf2 signaling. A phase I clinical trial in children and young adults with FA suggested potential hematologic benefits, further highlighting the translational relevance of targeting redox imbalance in FA [28].

To better understand the antioxidant state in FA, this study aims to analyze the profile of RBCs from patients homozygous for the *FANCA* c.295C>T variant, FA carriers heterozygous for the same variant, and healthy controls. Specifically, GSH levels and the activities of catalase, SOD, GPx, and GSTP1 were measured at baseline and after culture with and without the OS-related DEB exposure.

## 2. Materials and Methods

### 2.1. Subjects

Peripheral blood samples were collected from 7 patients with a clinical and genetic diagnosis of FA, all carrying the homozygous *FANCA* c.295C>T variant (3 males and 4 females, mean age 15.5 ± 10.9 years), and 9 relatives of FA patients (4 males and 5 females, mean age 28.4 ± 8.1 years), previously identified as heterozygous carriers for the *FANCA* c.295C>T variant. For comparative purposes, 9 healthy individuals (3 males and 6 females, mean age 23.3 ± 5.4 years) were recruited among volunteer blood donors from the Service of Immunohemotherapy, Unidade Local de Saúde Santo António.

Informed consent was obtained from all participants. All data were processed anonymously, in accordance with the Declaration of Helsinki, as revised in 2024.

### 2.2. Cells and Cell Cultures

From each participant, 10 mL of heparinized peripheral blood was collected by venipuncture and used for both cytogenetic analysis and enzymatic activity assessment in RBCs.

Whole blood cultures were established in Roswell Park Memorial Institute (RPMI)-1640 complete medium supplemented with 15% heat-inactivated fetal bovine serum and antibiotics (Gibco, Thermo Fisher Scientific, Waltham, MA, USA). Cultures were stimulated with 5 µg/mL phytohemagglutinin (PHA, Gibco, Thermo Fisher Scientific, USA) and incubated at 37 °C in a humidified atmosphere containing 5% CO_2_ for 72 h.

In the appropriate cultures, DEB (Sigma-Aldrich, St. Louis, MO, USA) was added at a final concentration of 0.5 µg/mL, 24 h after culture initiation, and cells were exposed for 48 h. Given that DEB is a suspected carcinogen, all procedures were performed under appropriate safety precautions.

RBC suspensions were obtained from whole blood and cultures after centrifugation at 2000 rpm and rinsed twice with RPMI medium. The purity of isolated RBCs was confirmed by microscopic examination of Wright-stained preparations in a Neubauer chamber, which revealed erythrocytes with no detectable leukocyte or platelet contamination.

### 2.3. Cytogenetic Analysis

After 72 h of culture, cells were incubated with 4 µg/mL colchicine (Gibco, Thermo Fisher Scientific, Waltham, MA, USA) for 1 h, followed by hypotonic treatment with 75 mM KCl (Sigma-Aldrich, St. Louis, MO, USA) and fixation in a 1:3 acetic acid:methanol solution (Fisher Chemical, Fair Lawn, NJ, USA). Chromosome preparations were obtained by air-drying and stained with 4% Giemsa (Sigma-Aldrich, St. Louis, MO, USA).

For each condition, 100 metaphases were analyzed by two independent scorers. To minimize selection bias, consecutive metaphases with intact and well-defined morphology were examined. Each cell was evaluated for chromosome number as well as for the presence and type of aberrations. Achromatic lesions narrower than one chromatid were scored as gaps, whereas those wider than one chromatid were considered breaks. Radial figures, dicentric and ring chromosomes, were scored as rearrangements with two or more breaks. Gaps were excluded from the calculation of chromosome aberration frequencies. Results of CI were expressed as the percentage of aberrant cells and the mean number of breaks per cell, the latter being the discriminative parameter for the diagnosis of Fanconi anemia.

### 2.4. GSH Content

The total GSH content of RBCs was determined by the DTNB–GSSG reductase recycling assay, as previously described [29]. Briefly, 100 µL of RBCs aliquots were precipitated with an equal volume of perchloric acid (5% final concentration) and centrifuged at 13,000 rpm for 10 min at 4 °C. After centrifugation, 100 µL acidic supernatant was neutralized with 100 µL 0.76 M KHCO_3_, followed by centrifugation at 13,000 rpm for 1 min.

A fresh reagent solution was prepared daily, containing 0.69 mM NADPH and 4 mM DTNB in 72 mM phosphate buffer. To measure total glutathione, 100 µL of sample, standard, or blank was added in duplicate to 96-well microplates, followed by 65 µL of freshly prepared reagent. Plates were incubated at 30 °C for 10 min in a plate reader (PowerWaveX; BioTek Instruments, Winooski, VT, USA), after which 40 µL of glutathione reductase (10 IU/mL in phosphate buffer) was added to each well.

The stoichiometric formation of 5-thio-2-nitrobenzoic acid (TNB) was monitored for 2 min at 415 nm and compared with a standard curve of GSH.

### 2.5. Enzymatic Activities

#### 2.5.1. Catalase

According to Aebi [30], catalase activity was determined by monitoring the decomposition of hydrogen peroxide (H_2_O_2_) at 240 nm in hemolyzed RBCs samples. Results were expressed as μmol of H_2_O_2_ decomposed per minute per mg of protein.

#### 2.5.2. Superoxide Dismutase (SOD)

The cytosolic Copper/zinc superoxide dismutase (CuZnSOD) and the mitochondrial manganese superoxide dismutase (MnSOD) activities were determined using the method of Flohé and Otting [30], with minor modifications. Superoxide radicals (O_2_•–) were generated by a xanthine–xanthine oxidase system, and the reduction in nitroblue tetrazolium (NBT) was monitored at 560 nm for 2 min. Potassium cyanide (2 mM) was added to inhibit CuZnSOD, enabling the specific measurement of MnSOD activity. Enzyme activity was expressed as units per milligram of protein, where one unit corresponds to the amount of enzyme required to inhibit the rate of NBT reduction by 50%.

#### 2.5.3. Glutathione Peroxidase (GPx)

GPx activity was measured using a coupled assay that monitors NADPH consumption, reflecting the rate of GSSG formation during the GPx-catalyzed reaction, as described previously [31]. The final reaction mixture contained 1 mM GSH, 0.24 U/mL glutathione reductase, 0.15 mM NADPH, 0.15 mM H_2_O_2_, 1 mM EDTA, 1 mM sodium azide (NaN_3_), and 1 mM potassium phosphate buffer (pH 7.0). After a 5 min preincubation at 25 °C, the reaction was initiated by adding H_2_O_2_ (1.5 mM final concentration), and the decrease in NADPH absorbance was monitored at 340 nm over 4 min. Nonenzymatic background rates (buffer instead of enzyme source) were subtracted. GPx activity was expressed as nmol NADPH oxidized per minute per mg of protein, using an extinction coefficient for NADPH of 6.22 mM^−1^·cm^−1^.

#### 2.5.4. Pi-Class Glutathione S-Transferase (GSTP1)

GSTP1 activity was measured by monitoring the formation of the conjugate between reduced GSH and 1-chloro-2,4-dinitrobenzene (CDNB) at 340 nm in hemolyzed samples, as described by Habig et al. [32]. Activity was calculated using the extinction coefficient of 9.6 mM^−1^·cm^−1^ and expressed as µmol of conjugate formed per minute per mg of protein.

### 2.6. Statistical Analysis

Statistical analysis was performed using GraphPad Prism 9.0.0 (GraphPad Software, San Diego, CA, USA). Normality of the data was assessed using the Shapiro–Wilk test. For comparisons of basal GSH levels and antioxidant enzyme activities between groups, one-way ANOVA was applied, followed by Dunnett’s multiple comparisons test against controls. To evaluate the effect of culture and DEB-induced oxidative stress, two-way ANOVA was performed, followed by Tukey’s multiple comparisons test. Correlation analyses between erythroid parameters and GSH levels or antioxidant enzyme activities were performed using Spearman’s rank correlation test. A significance level of *p* = 0.05 was considered.

## 3. Results

### 3.1. Evaluation of Chromosome Instability in Study Groups

The diagnostic classification of each study group was confirmed by CI analysis using the DEB sensitivity test, according to the internal reference thresholds established at the Laboratory of Cytogenetics, of School of Medicine and Biomedical Sciences, for the number of mean breaks per cell (negative ≤0.28 and positive ≥0.96) (Table 1).

FA patients had high levels of CI, with an increased percentage of aberrant cells (86.39 ± 14.18%) and a higher mean number of breaks per cell (6.75 ± 4.24), substantially above our internal positive diagnostic threshold.

Controls showed low levels of CI (5.76% ± 5.91 aberrant cells; 0.01 ± 0.07 breaks per cell), within the internal negative threshold. Similar results were observed in FA carriers (3.79 ± 2.48% aberrant cells; 0.04 ± 0.03 breaks per cell).

Overall, high CI was specific to FA patients, supporting the group classification.

### 3.2. Basal GSH Levels and Antioxidant Enzyme Activities in RBCs

**Figure 1:**
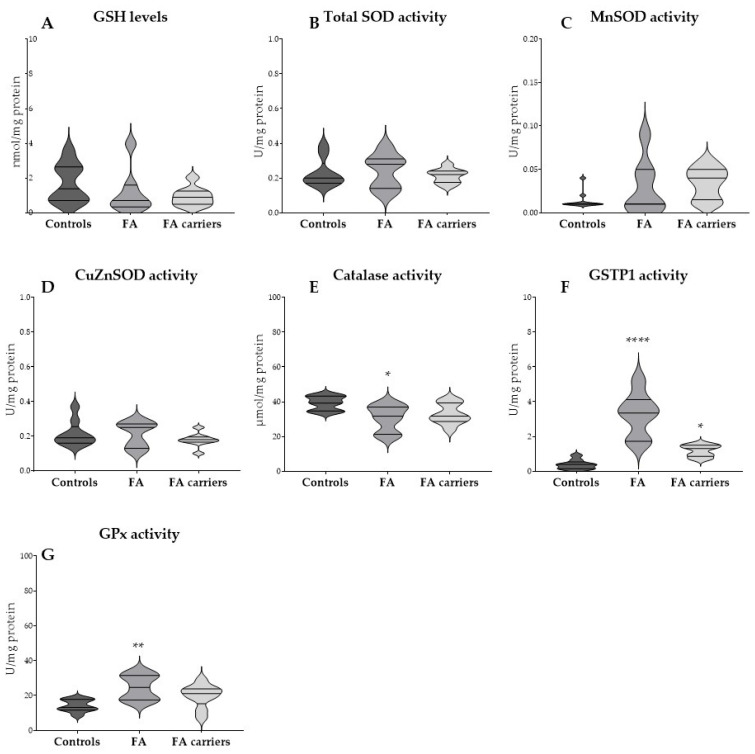
Basal glutathione (GSH) levels and antioxidant enzyme activities in red blood cells (RBCs). Violin plots represent the distribution of (**A**) GSH, (**B**) total superoxide dismutase (SOD), (**C**) Manganese superoxide dismutase (MnSOD), (**D**) Copper/zinc superoxide dismutase (CuZnSOD), (**E**) catalase, (**F**) Pi-class glutathione S-transferase (GSTP1), and (**G**) glutathione peroxidase (GPx) activities in RBCs from controls, Fanconi anemia (FA) patients, and FA carriers. Data was analyzed by one-way ANOVA followed by Dunnett’s multiple comparisons test. * *p* < 0.05, ** *p* < 0.01, **** *p* < 0.0001.

Basal GSH levels and the activities of catalase, SOD (total and isoforms), GPx, and GSTP1 were evaluated in RBCs from FA patients, FA carriers and healthy controls (Figure 1).

No significant differences were observed in basal GSH levels and total SOD among the three groups (Figure 1A,B). When the two SOD isoforms were analyzed separately, neither MnSOD nor CuZnSOD activities exhibited significant differences (Figure 1C,D), although RBCs from FA patients and FA carriers showed a tendency towards increased MnSOD activity compared to controls where activity was mostly close to zero.

Significant differences were observed in catalase, GSTP1, and GPx activities. Catalase activity was significantly reduced in RBCs from FA patients compared to controls (*p* = 0.0152), while RBCs from FA carriers showed intermediate levels that did not differ significantly (Figure 1E). GSTP1 activity was significantly higher in RBCs from FA patients compared to controls (*p* < 0.0001). RBCs from FA carriers also showed increased activity, though to a lesser extent (*p* = 0.0470) (Figure 1F). GPx activity was significantly higher in RBCs from FA patients compared to controls (*p* = 0.0034), whereas RBCs from FA carriers showed intermediate levels that did not reach statistical significance (Figure 1G).

### 3.3. Erythroid Parameters and MnSOD Activity from FA Patients

To further investigate the increased MnSOD activity observed in FA erythrocytes, we analyzed erythroid parameters from hemograms of FA patients at the time of sample collection, which are summarized in Table 2.

**Figure 2:**
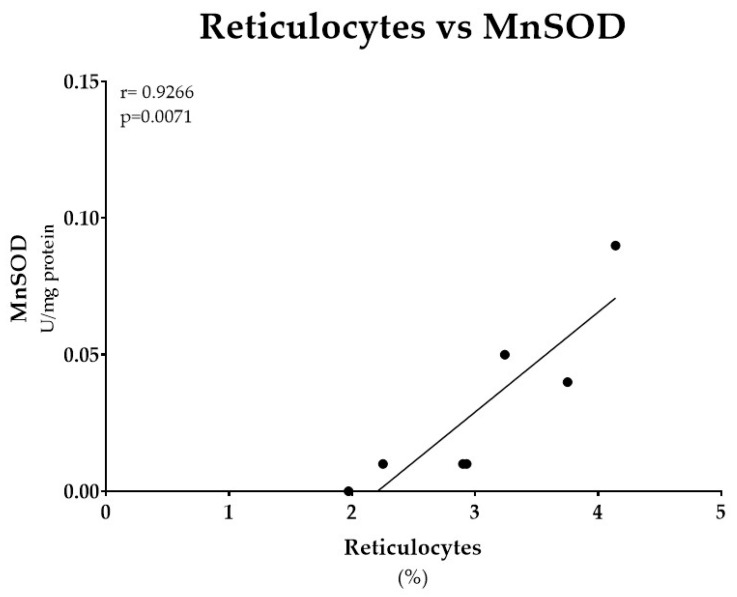
Correlation between reticulocyte percentage and MnSOD activity in red blood cells (RBCs) from FA patients. A significant positive correlation was observed (r = 0.93, *p* = 0.0167).

All evaluated erythroid parameters were altered in FA patients. Hemoglobin (Hb) concentrations were consistently below the reference range, ranging from 7.1 to 13.8 g/dL, and were paralleled by reduced red blood cell (RBC) counts, 1.9 to 4.6 ×10^6^/µL. Mean corpuscular volume (MCV), 96.6 to 117.8 fL, and red cell distribution width (RDW), 15.1 to 21.1%, were increased in all patients. Fetal hemoglobin (HbF) was elevated in several cases, reaching 12.7%. Reticulocyte counts were consistently increased, 1.97 to 4.14%. A significant positive correlation was observed between reticulocyte counts and MnSOD activity in RBCs from FA patients, r = 0.93, *p* = 0.0167 (Figure 2). No other significant correlations were identified between erythroid parameters and GSH levels and antioxidant enzyme activities (Supplementary Table S1).

### 3.4. GSH Levels and Antioxidant Enzyme Activities in RBCs After Culture and the Effect of DEB-Toxicity

**Figure 3:**
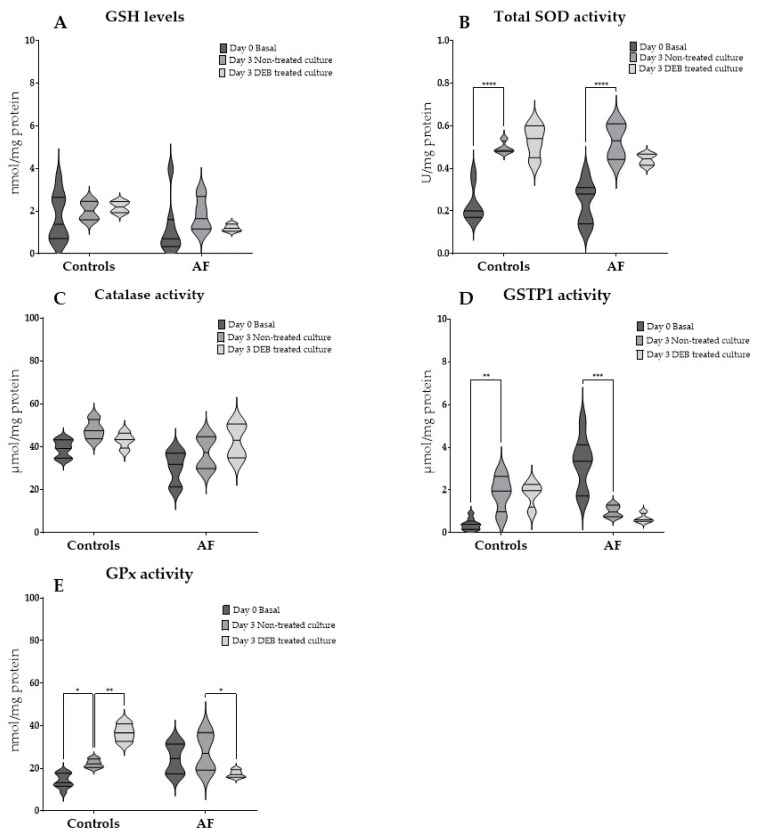
Glutathione (GSH) levels and antioxidant enzyme activities in red blood cells (RBCs) after cultures with and without 1,2,3,4-Diepoxybutane (DEB). Violin plots represent the distribution of (**A**) GSH, (**B**) total superoxide dismutase (SOD), (**C**) catalase, (**D**) Pi-class glutathione S-transferase (GSTP1), and (**E**) glutathione peroxidase (GPx) activities at Day 0 Basal, after 3 days of culture without treatment (Day 3 Non-treated culture), and after 3 days of culture followed by DEB exposure (Day 3 DEB Treated culture). Data were analyzed by two-way ANOVA followed by Sidak’s multiple comparisons test. * *p* < 0.05, ** *p* < 0.01, *** *p* < 0.001, **** *p* < 0.0001.

To assess the effects of culture and OS induced by DEB, GSH levels and antioxidant enzyme activities were measured in RBCs from controls and FA patients at baseline (day 0 Basal), after 3 days of culture without treatment (Day 3 non-treated culture), and after DEB exposure (Day 3 DEB treated culture) (Figure 3).

Culture and OS induced by DEB had no significant effect on GSH levels in RBCs both from FA patients and controls (Figure 3A).

Total SOD activity increased significantly after culture in RBCs from both controls (*p* < 0.0001) and FA patients (*p* < 0.0001) (Figure 3B). However, after DEB exposure, no significant differences were observed when compared to non-treated cultures.

Catalase activity did not change significantly after culture or DEB exposure in either group (Figure 3C).

GSTP1 activity exhibited opposite responses between groups (Figure 3D,E). In RBCs from controls, culture significantly increased GSTP1 activity (*p* = 0.0066), but no additional differences were observed after DEB exposure. In contrast, RBCs from FA patients showed a significant reduction in GSTP1 activity after culture (*p* = 0.0001), but no additional significant differences were observed after DEB exposure. GPx activity also exhibited opposite responses between groups (Figure 3E). In RBCs from controls, GPx activity increased significantly after culture (*p* = 0.0451) and was further elevated after DEB exposure (*p* < 0.0001). On the contrary, in RBCs from FA patients, no significant differences were observed after culture, but a significant decrease occurred upon DEB exposure (*p* = 0.0308).

## 4. Discussion

This study provides new insights into the redox state of FA through the analysis of the antioxidant profile of RBCs in individuals with the homozygous *FANCA* c.295C>T variant. Our findings suggest that FA RBCs exhibit a complex and impaired antioxidant response that, although compensated at baseline, fails to establish an effective defense against oxidative and genotoxic stress.

Contrary to previous studies describing reduced antioxidant capacity in FA [7,33,34], our findings showed no significant differences in RBCs basal GSH levels and in the activities of total SOD, MnSOD, and CuZnSOD between FA patients, FA carriers, and controls. It is worth noting that FA patients and carriers showed a tendency towards increased mitochondrial MnSOD activity. Knowing that during the process of RBC maturation mitochondria are lost, this finding suggests the presence of immature erythroid cells in peripheral blood, which could be consistent with the stress erythropoiesis typically observed in FA [24,35]. To support this interpretation, hematological data from the time of sample collection showed increased reticulocyte counts, which correlated positively with MnSOD activity. Together with the consistently increased RDW values, elevated HbF levels, and macrocytosis reflected by increased MCV, these results provide direct evidence of abnormal stress erythropoiesis in FA. This is in line with previous reports highlighting RDW as a biomarker of ineffective erythropoiesis in FA [24]. Interestingly, recent studies have shown that residual mitochondria can persist in mature RBCs under pathological conditions, such as sickle cell disease, where they contribute to oxidative imbalance and altered redox signaling [36,37]. In this context, the increased MnSOD activity observed in FA could reflect not only the presence of immature erythroid cells, but also abnormal mitochondrial clearance and redox regulation. Mitochondrial dysfunction is a hallmark of FA, and there is evidence that impaired mitochondrial redox balance contributes to defective erythropoiesis, RBC loss, and bone marrow failure [9,12].

RBCs’ basal catalase activity was significantly reduced in FA patients. This observation is consistent with earlier studies reporting reduced catalase activity in FA erythrocytes and with evidence that impaired catalase function is associated with increased CI and oxidative DNA lesions such as 8-hydroxydeoxyguanosine [21,38,39].

For the first time, it is shown that GSTP1 and GPx activities are significantly increased in RBCs from FA patients, with GSTP1 also being upregulated in RBCs from FA carriers. This intermediate phenotype, between FA and controls, aligns with previous studies that reported subtle redox alterations in female FA carriers, although in our population, no differences were observed between sexes [40]. These new findings suggest a state of endogenous oxidative preconditioning, in which the specific antioxidant enzymes GSTP1 and GPx, are upregulated in response to continuous redox imbalance, making them potential biomarkers to assess long-term exposure to OS-related toxicity.

The analysis of GSH levels and catalase activity under culture and DEB exposure, a potent alkylating agent, showed no different responses between FA patients and controls, which suggests a similar protective response upon genotoxicity induced by DEB. Total SOD activity increased significantly both in cultured RBCs from FA patients and controls, which was maintained but not further enhanced after DEB exposure. It should be noted that cell culture itself can act as a source of OS, which may account for some of the observed changes in enzyme activities. Despite these significant changes, no differences were observed between groups, suggesting that SOD function is not impaired in FA. By contrast, GSTP1 and GPx activities showed opposite responses between the two groups. In controls, GSTP1 activity increased after culture and remained elevated upon DEB exposure, while GPx activity was induced by culture and further upregulated after exposure to DEB. This pattern is consistent with a robust and sustained antioxidant defense. GSTP1 catalyzes the conjugation of glutathione to electrophilic compounds, including reactive aldehydes, thereby preventing their accumulation and protecting proteins and membrane lipids from oxidative damage [18]. GPx complements this defense by acting as a secondary barrier to hydrogen peroxide detoxification and by reducing both small organic peroxides and complex lipid hydroperoxides, thereby protecting Hb and membrane structures from oxidative damage [15,41]. In FA RBCs, however, GSTP1 activity decreased significantly after culture and remained decreased under DEB exposure, whereas GPx activity was not affected by culture but declined significantly after DEB exposure. These responses highlight a fundamental breakdown in the adaptive antioxidant capacity of FA RBCs. While this pattern may reflect enzyme exhaustion or impaired regulation despite unchanged glutathione availability, alternative mechanisms such as post-translational modifications, increased protein degradation, or impaired substrate recycling cannot be excluded.

These new findings extended previous studies showing the role of hemoglobin content, particularly HbF, which displays higher activities of antioxidant enzymes such as GST, catalase, and SOD, in protection against DEB-induced toxicity in primary lymphocytes [41,42,43,44]. It is noteworthy that, in FA patients, progression to bone marrow failure is typically preceded by increased levels of HbF-enriched RBCs and macrocytosis, reflecting stress erythropoiesis [45]. With this study, it is hypothesized that, in addition to decreased Hb content, the impaired coordination of glutathione-dependent antioxidant enzymes is an additional factor contributing to the reduced protective capacity of FA RBCs. More broadly, these findings reinforce that redox dysregulation in FA is not confined to DNA repair-deficient cells but is already evident in circulating RBCs, highlighting the impact of systemic oxidative stress and abnormal stress erythropoiesis as key pathogenic features [24,46]. This supports the view that RBC antioxidant profiles could serve as accessible biomarkers of disease severity or therapeutic response in situations with increased genotoxicity [47].

Despite the new insights into FA redox biology, certain limitations should be considered. In addition to the limited sample size, which reflects the rarity of FA, all patients included in this study carried the same homozygous *FANCA* c.295C>T variant. Nevertheless, this provides a genetically homogeneous cohort that minimizes variability and strengthens the reliability of the observed alterations. As FA is a genetically heterogeneous disorder, further comprehensive studies including individuals from other complementation groups are essential to expand the current knowledge and to clarify if different genetic backgrounds may influence redox biology in FA. Moreover, since mature RBCs lack active transcription, we cannot exclude the contribution of transcriptional dysregulation in precursor cells or peripheral leukocytes to the differences observed in antioxidant enzyme activities. Future studies addressing redox gene expression in these compartments would therefore provide important complementary information.

From a translational perspective, these results strengthen the rationale for antioxidant-based interventions in FA whenever a situation of increased OS-related genotoxicity is present. Compounds such as NAC, ALA, and quercetin have shown protective effects on genomic stability in preclinical and early clinical studies [26,27,28], while activation of sirtuin pathways may enhance mitochondrial resilience [48,49]. However, strategies must be applied cautiously given the dual role of ROS in physiological signaling.

## 5. Conclusions

This work shows that RBCs from FA patients carrying the *FANCA* c.295C>T variant display an impaired antioxidant response. So far, this is the first study showing upregulation of GSTP1 and GPx activities at baseline, suggesting an adaptive state to endogenous OS, and that this compensation collapses under increased OS conditions. These dysfunctional dynamics occur despite unchanged glutathione availability, pointing to a loss of enzymatic coordination rather than substrate depletion.

These new findings highlight a systemic redox imbalance in FA, possibly consistent with mitochondrial dysfunction and abnormal stress erythropoiesis as key pathogenic features. Notably, the study suggests GSTP1 activity as a central biomarker in FA RBCs, which is severely compromised under increased oxidative and genotoxic challenge. This mechanistic insight supports the rationale for therapeutic interventions targeting oxidative stress in the comprehensive management of Fanconi anemia. Antioxidant-based strategies to restore coordinated response between cytosolic and mitochondrial defenses may also hold translational value in disease management.

## Figures and Tables

**Table 1:** Chromosome instability in 1,2,3,4-diepoxybutane (DEB)-treated (0.5 µg/mL) lymphocytes obtained from peripheral blood cultures.

	Controls		FA		FA Carriers	
	% Aberrant Cells	Mean No Breaks/Cell	% Aberrant Cells	Mean No Breaks/Cell	% Aberrant Cells	Mean No Breaks/Cell
	1.00	0.01	85.70	3.14	2.10	0.02
	18.20	0.20	98.00	14.36	1.00	0.01
	1.00	0.01	83.30	2.70	4.00	0.05
	13.8	0.19	55.00	4.20	5.00	0.07
	6.00	0.06	96.00	9.91	2.00	0.03
	6.00	0.06	100.00	9.74	4.00	0.04
	3.80	0.05	86.70	3.18	3.00	0.03
	1.00	0.01			10.00	0.11
	1.00	0.01			3.00	0.04
Mean ± SD	5.76 ± 5.91	0.01 ± 0.70	86.39 ± 14.18	6.75 ± 4.24	3.79 ± 2.48	0.04 ± 0.003

**Table 2:** Erythroid parameters of FA patients at the time of sample collection.

	RBC (×10^6^/µL)	Hb (g/dL)	HbF (%)	MCV (fL)	RDW (%)	Reticulocytes (%)
	[4.00–5.20]	[11.10–14.10]	[<2.00%]	[77.00–95.00]	[11.60–14.00]	[1.00–2.00%]
FA1	3.74	9.50	12.70	104.10	19.20	3.24
FA2	3.00	10.60	--	103.80	17.30	4.14
FA3	4.60	12.20	1.20	105.20	14.70	2.25
FA4	4.17	13.80	--	96.60	15.40	2.90
FA5	2.10	7.70	12.60	117.80	18.90	3.75
FA6	1.90	7.10	8.10	109.00	21.10	2.93
FA7	2.67	8.60	--	97.00	15.10	1.97

Red blood cell count (RBC); Hemoglobin (Hb); Fetal hemoglobin (HbF); Mean Corpuscular Volume (MCV); Red cell distribution width (RDW). Reference ranges are indicated in brackets.

## Data Availability

The raw data supporting the conclusions of this article will be made available by the authors upon reasonable request.

## Supplementary Materials

The following supporting information can be downloaded at: https://www.mdpi.com/article/10.3390/antiox14101150/s1, Table S1: Correlation analysis between erythroid parameters and GSH levels or antioxidant enzyme activities.

## Abbreviations

The following abbreviations are used in this manuscript:

ALA	α-lipoic acid
BMF	Bone marrow failure
CDNB	1-chloro-2,4-dinitrobenzene
CI	Chromosome instability
CuZnSOD	Copper/zinc superoxide dismutase
DEB	1,2,3,4-diepoxybutane
FA	Fanconi anemia
GPx	Glutathione peroxidase
GSH	Glutathione
GST	Glutathione *S*-transferase
GSTP1	Pi-class glutathione *S*-transferase
Hb	Hemoglobin
HbF	Fetal hemoglobin
MnSOD	Manganese superoxide dismutase
NAC	*N*-acetylcysteine
NADH	Nicotinamide Adenine Dinucleotide Phosphate
NBT	Nitroblue tetrazolium
OS	Oxidative stress
RBC	Red blood cell count
RBCs	Red blood cells
RDW	Red cell distribution width
RPMI	Roswell Park Memorial Institute
ROS	Reactive oxygen species
SOD	Superoxide dismutase
