# Supplementary material for: Red Blood Cell Antioxidant State in Fanconi Anemia: The Highlighted Roles of Pi-Class Glutathione S-Transferase and Glutathione Peroxidase
Source: Antioxidants (Basel). 2025 Sep 23;14(10):1150. doi: 10.3390/antiox14101150 (PMC12561454; doi:10.3390/antiox14101150)
Supplement: Supplementary file 1 [file antioxidants-14-01150-s001.zip › antioxidants-3895830-supplementary.pdf]

Table S1. Correlation analysis between erythroid parameters and GSH levels or antioxidant enzyme activities

	**RBC (x106/µl)** **[4.00-5.20]**	**Hb (g/dL)** **[11.10-14.10]**	**HbF (%)** **[<2.00%]**	**MCV (fL)** **[77.00-95.00]**	**RDW (%)** **[11.60-14.00]**	**Reticulocytes (%)** **[1.00-2.00%]**
GSH	r= -0.1786 p= 0.9635	r= -0.0357 p= 0.9635	r= 0.8000 p= 0.3330	r= 0.0714 p= 0.9063	r= 0.4643 p= 0.3024	r= 0.6710 p= 0.1667
Total SOD	r= 0.2857 p= 0.5560	r= 0.2143 p= 0.6615	r= 0.8000 p= 0.3330	r= 0.2143 p= 0.6615	r= 0.2857 p= 0.5560	r= 0.7500 p= 0.0663
MnSOD	r= 0.0370 p= 0.9738	r= 0.0741 p= 0.8952	r= 0.9487 p= 0.1667	r= 0.2224 p= 0.6452	r= 0.4447 p= 0.3286	**r= 0.9266 p**= 0.0071**
CuZnSOD	r= 0.5000 p= 0.22667	r= 0.4643 p= 0.3024	r= -0.2000 p= 0.9167	r= 0.1429 p= 0.7825	r= -0.1071 p= 0.8397	r= 0.5714 p= 0.2000
Catalase	r= 0.5357 p= 0.2357	r= 0.5000 p= 0.2667	r= -0.400 p= 0.7500	r= 0.2143 p= 0.6615	r= -0.5000 p= 0.2667	r= 0.2143 p= 0.6615
GSTP1	r= 0.1429 p= 0.7825	r= 0.1429 p= 0.7825	r= 0.6000 p= 0.5948	r= 0.2500 p= 0.5948	r= 0.3214 p= 0.4976	r= 0.6429 p= 0.1389
GPx	r= -0.2857 p= 0.5560	r= -0.2143 p= 0.6615	r= -0.2000 p= 0.9167	r= 0.1071 p= 0.8397	r= -0.0743 p= 0.0963	r= -0.2857 p= 0.1389

Red blood cell count (RBC); Hemoglobin (Hb); Fetal hemoglobin (HbF); Mean Corpuscular Volume (MCV); Red cell distribution width (RDW). Reference ranges are indicated in brackets. ** $p < 0.01$.
